# 15-LOX metabolites and angiogenesis: angiostatic effect of 15(S)-HPETE involves induction of apoptosis in adipose endothelial cells

**DOI:** 10.7717/peerj.635

**Published:** 2014-10-21

**Authors:** Sasikumar J. Soumya, Sheela Binu, Antony Helen, Pallu Reddanna, Perumana R. Sudhakaran

**Affiliations:** 1 Department of Biochemistry, University of Kerala, Thiruvananthapuram, Kerala, India; 2 National Institute of Animal Biotechnology, University of Hyderabad, Hyderabad, India; 3 Department of Computational Biology and Bioinformatics, University of Kerala, Kariavattom, Thiruvananthapuram, Kerala, India; 4 Inter-University Centre for Genomics and Gene Technology, University of Kerala, Kariavattom, Thiruvananthapuram, Kerala, India

**Keywords:** Adipose tissue-angiogenesis, 15-lipoxygenase, 15(S)-HPETE, VEGF, Apoptosis

## Abstract

Inflammation is critical in the dysregulated growth of adipose tissue and associated vascular dysfunctions. 15-Lipoxygenase metabolites, important mediators of inflammation in adipose tissue during obese conditions, may contribute to codependence of inflammation and angiogenesis in adipose tissue. We have already reported the pro-angiogenic effect of 15(S)-HETE in adipose tissue. The present study was designed to understand the effect of 15(S)-HPETE, precursor of 15(S)-HETE, on angiogenesis in adipose tissue. Results showed that 15(S)-HPETE exerts an anti-angiogenic effect in adipose tissue. This was evidenced from decreased endothelial sprouting in adipose tissue explants, inhibition of angiogenic phenotype in adipose endothelial cells, decreased production of CD31 and VEGF in endothelial cells treated with 15(S)-HPETE. Further studies to examine the molecular mechanism of anti-angiogenic effect of 15(S)-HPETE showed that it inhibited cell survival signaling molecule Akt and anti-apoptotic Bcl-2 and also activated caspase-3 in adipose endothelial cells. These observations indicate that 15(S)-HPETE exerts its angiostatic effect in adipose tissue by inducing apoptosis of endothelial cells.

## Introduction

15-Lipoxygenase (15-LOX) and its metabolites seem to exhibit both pro-inflammatory and anti-inflammatory activities. The transfection of 15-LOX to rat kidney suppresses inflammation (Munger et al., 1999). In a murine model of airway inflammation, it was reported that the lungs of 12/15-LOX deficient mouse had more pronounced inflammatory responses than those of control mouse (Conrad et al., 1992). 15-LOX isoforms also possess opposing effects on cancer cells, being the isoform 1 stimulatory and the isoform 2 inhibitory of cancer cell proliferation (His, Wilson & Eling, 2002). Thus 15-LOX exhibits differential effects on various inflammatory disorders. Our previous study showed that 15-LOX metabolites possess opposing effects on angiogenesis (Soumya et al., 2012). 15(S)-HETE induces pro-angiogenic response and 15(S)-HPETE induces anti-angiogenic response in Human Umbilical Vein Endothelial Cells (HUVECs). Further, the pro-angiogenic effect of 15(S)-HETE is reversed by 15(S)-HPETE and vice versa suggesting that, one of the factors contributing to the differential effects of 15-LOX metabolites on angiogenesis is the relative levels of 15(S)-HETE and 15(S)-HPETE (Soumya et al., 2012).

Angiogenesis that accompanies chronic inflammation tends to prolong and intensify the inflammatory response (Granger & Senchenkova, 2010). The codependence of angiogenesis and inflammation is a pivotal point in the expansion of adipose tissue and the development of obesity. In response to inflammatory signals, adipocytes induce expression of inflammatory mediators including TNF-*α*, plasminogen activator inhibitor-1, IL-1*β*, IL-6, IL-8, IL-10, IL-5, eicosanoids, leptin, adiponectin and resistin (Feng, Zhang & Xu, 2013). In addition to these, lipid mediators including Cyclooxygenase (COX) and Lipoxygenase (LOX) metabolites [prostaglandins, thromboxane, leukotrienes, lipoxins, epoxyeicosatrienoic acids, other fatty acid epoxides, hydroxyeicosatetraenoic acids], platelet-activating factor, lysophosphatidic acid, sphingosine-1-phosphate, 2-arachidonoyl glycerol and other lipid amides also modulate low grade inflammation in adipose tissue (Iyer et al., 2010).

Lipoxygenase metabolites are important lipid mediators in obesity and adipocyte dysfunction (Cole et al., 2012; Dobrian et al., 2010; Ye, 2009). Lipoxygenase pathway involves the conversion of arachidonate to 5-, 12- or 15-hydroperoxyeicosatetraenoic acids (HPETEs) by 5-, 12- or 15-lipoxygenase respectively. HPETEs are metabolized to hydroxyeicosatetraenoic acids (HETEs) by the enzyme, glutathione peroxidase. Pro-angiogenic effect of 15(S)-HETE in human dermal microvascular endothelial cells and retinal microvascular endothelial cells has been reported (Kundumani-Sridharan et al., 2010; Bajpai et al., 2007; Zhang, Cao & Rao, 2005). We also observed that 15(S)-HETE induced angiogenesis in stromovascular fractions of adipose tissue (Soumya et al., 2013). The effect of 15(S)-HPETE, precursor of 15(S)-HETE, on angiogenesis in adipose tissue is not clear. Therefore the objective of the present study was to examine whether 15(S)-HPETE affects angiogenesis in adipose tissue. The results presented here indicate that 15(S)-HPETE causes anti-angiogenic effect in stromovascular fractions of adipose tissue by induction of apoptosis in endothelial cells.

## Materials

Collagenase, MCDB 131 medium, Eagle’s Minimum Essential Medium (MEM), antibiotic antimycotic solution, protease inhibitor cocktail, bovine serum albumin, o-Phenylene diamine dihydrochloride, antibodies against VEGF, CD31, Bcl-2, Bax, protein kinase B*α*, phospho serine, HRP conjugated secondary anti mouse IgG, anti goat IgG, anti rabbit IgG, protein A agarose, RNA isolation kit and caspase-3 assay kit were purchased from M/s Sigma Aldrich Co., USA. Cell culture plastic wares and ELISA plates were purchased from NUNC, Denmark. 15(S)-HPETE was prepared by incubating arachidonic acid with 15-LOX, as described earlier (Mahipal et al., 2007).

## Methods

### Angiogenesis assay using adipose tissue explants

Angiogenesis assay was carried out by the procedure of Gealekman et al. (2008) with some modifications. 6–8 week old Sprague-Dawley rats were used in experiments and the rats were maintained in animal house, Department of Biochemistry, University of Kerala. 1 mm thick fat pad explants, prepared from freshly harvested rat epididymal fat pads, were maintained in MCDB 131 medium at 37 °C in a 95% air and 5% CO_2_ atmosphere. Enzymatic digestion was performed in MEM supplemented with 1 mg/ml collagenase for 30 min at 37 °C. The undigested tissue comprising stromovascular fraction of adipose tissue was collected, washed, cut into small pieces, and used for the angiogenesis assay. Capillary sprouts from the fat pad explants were examined under a microscope (Leica).

### Isolation and culture of adipose endothelial cells

Endothelial cells were isolated by collagenase digestion of epididymal fat pads by the procedure of Gealekman et al. (2008) with some modifications. Before reaching confluence, the primary cultures of endothelial cells were subcultured up to 2 passages. The cells were maintained in culture at 37 °C in a 95% air and 5% CO_2_ atmosphere in a Sanyo carbon dioxide incubator in MCDB 131 medium supplemented with 10% FCS. All experiments were carried out as approved by the Institutional Animal Ethics Committee, Department of Biochemistry, University of Kerala, Thiruvananthapuram, India (approval no. IAEC-KU-10/08-09-BC-PRS(9)).

### Enzyme linked immunosorbent assay (ELISA)

Indirect ELISA was carried out using specific primary antibody and HRP conjugated secondary antibody (Engvall & Perlmann, 1971). Cell culture medium and cell lysate pre-coated onto ELISA plates served as antigens. o-Phenylene diamine dihydrochloride was used as the substrate. The concentration of antigen was estimated by measuring the absorbance of the colored HRP product at 490 nm in a multiwell microplate reader (Thermo Multiskan Spectrum). Protein was estimated by Lowry’s method (Lowry et al., 1951).

### Western Blot analysis

Western blotting was done according to the procedure of Towbin, Staehelin & Gordon (1979). Proteins were separated in a 7.5% polyacrylamide gel, transferred onto nitrocellulose membrane, and probed using specific primary antibody (dilution of 1:1,000) followed by secondary antibody (IgG-HRP) (dilution of 1:1,000). The bands were detected by staining with 3, 3′-diamino benzidine.

### Apoptotic assays

#### DAPI staining

DAPI staining was performed according to the protocol of Yim et al. (2000). Cells were fixed by incubation in 4% paraformaldehyde for 30 min. Following washing with PBS, the cells were incubated in DAPI solution for 30 min in dark. Stained cells were observed under a fluorescent microscope.

#### Assay of Caspase-3

Caspase-3 was assayed using a kit purchased from Sigma Chemical Co., USA following the protocol provided by the manufacturer. Briefly, the cell lysate prepared in the lysis buffer (50 mM HEPES, pH 7.4, 5 mM CHAPS, 5 mM DTT) was incubated with the peptide conjugate–substrate DEVD-*p*-nitroanilide (pNA) at 37 °C for 90 min. Intensity of the colored product *p*-nitroaniline was measured by an automated microplate reader at 405 nm.

### Statistical analysis

All the data were expressed as mean with standard error of mean. The statistical significance of difference was measured by Students *t*-test and one way ANOVA. A value of *p* < 0.05 was considered significant.

## Results

### Effect of 15(S)-HPETE on adipose tissue explants

**Figure 1:**
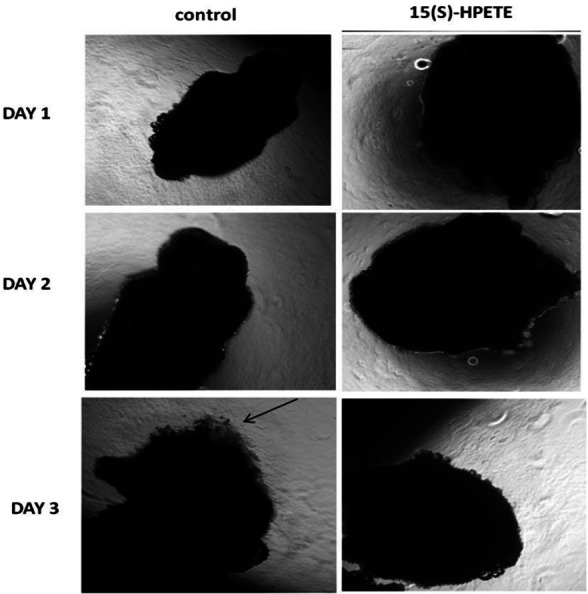
Effect of 15(S)-HPETE on adipose tissue explants. Adipose tissue explants were maintained in culture in MCDB 131 medium with and without 15(S)-HPETE (0.1 µM). The formation of endothelial sprouts from adipose tissue explants in culture was visualized and photographed every 24 h under a microscope (4x). Arrow indicates endothelial sprout.

To study the effect of 15(S)-HPETE on angiogenesis in adipose tissue, angiogenesis assay using adipose tissue explants was performed. Adipose tissue explants were maintained in culture in MCDB 131 medium in presence and absence of 15(S)-HPETE. Morphological analysis revealed that 15(S)-HPETE significantly inhibited the formation of endothelial sprouts from adipose tissue explants when compared to control (Fig. 1). In control explants, sprouting started to appear on the third day of culture, whereas no significant sprouting was seen in adipose tissue explants treated with 15(S)-HPETE during this period suggesting the inhibitory effect of 15(S)-HPETE on adipose tissue-angiogenesis.

### Effect of 15(S)-HPETE on adipose endothelial cells

**Figure 2:**
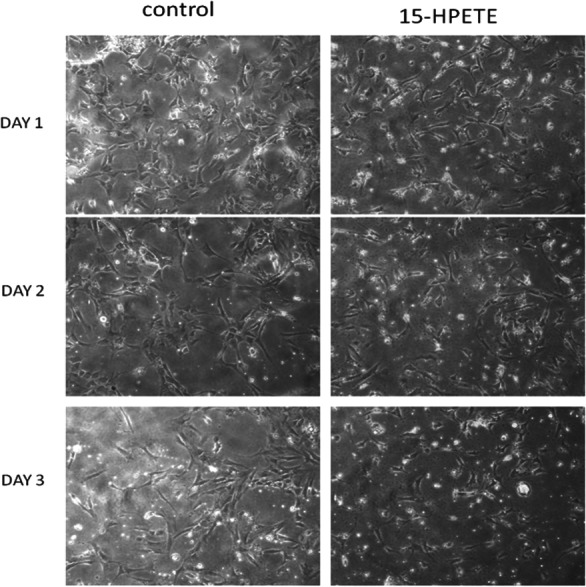
Effect of 15(S)-HPETE on adipose endothelial cells. Microvascular endothelial cells derived from epididymal adipose tissue of rats were maintained in culture in MCDB 131 medium supplemented with 15(S)-HPETE (0.1 µM). Cells treated with MCDB 131 containing 0.1% alcohol served as control. The cells were observed under an inverted microscope (10x) every 24 h and photographed.

The influence of 15(S)-HPETE on adipose endothelial cells was also studied. Endothelial cells derived from adipose tissue were maintained in culture in MCDB 131 medium treated with 15(S)-HPETE and observed every 24 h. Endothelial cells treated with 15(S)-HPETE did not show any significant morphological changes relevant to angiogenesis even after 72 h in culture whereas in control cells, grouping of cells started to appear during this period, confirming the inhibitory effect of 15(S)-HPETE on angiogenesis in adipose endothelial cells (Fig. 2).

### Effect of 15(S)-HPETE on the production of CD31

**Figure 3:**
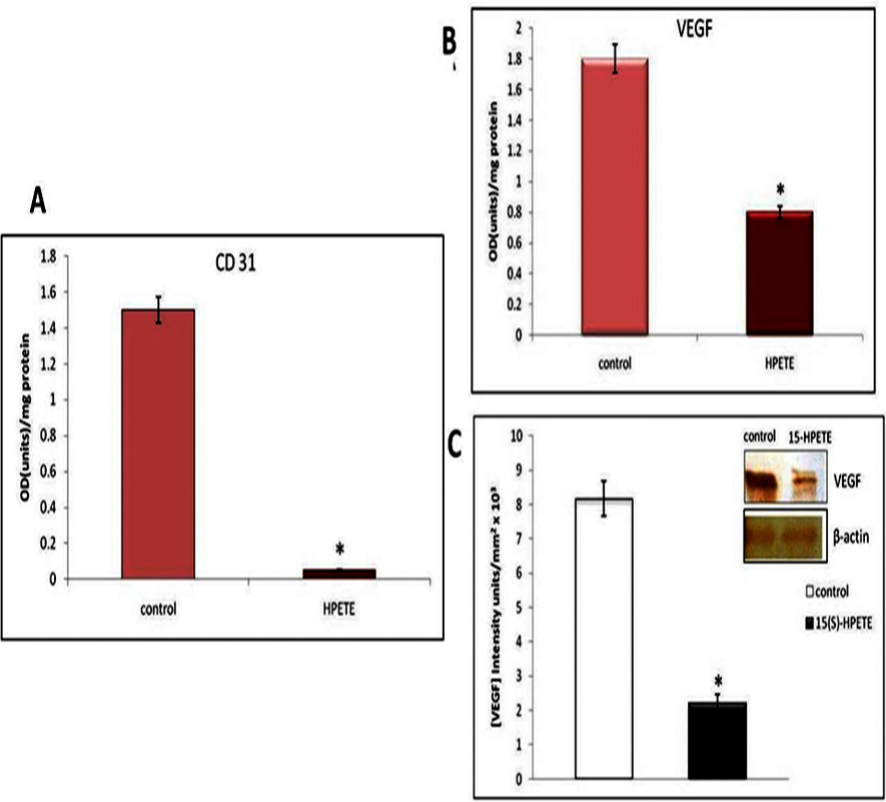
Effect of 15(S)-HPETE on the production of CD31 and VEGF. Microvascular endothelial cells derived from adipose tissue were maintained in culture in MCDB 131 medium containing 15(S)-HPETE (0.1 µM) for 48 h. Cells treated with MCDB 131 medium containing 0.1% alcohol served as control. (A) Cell layer was harvested and the level of cell associated CD31 was estimated by ELISA using anti-CD31. (B) Medium was collected and estimated the level of VEGF by ELISA. (C) Medium was collected, concentrated and subjected to Western blot using anti-VEGF as primary antibody and anti-goat IgG-HRP as secondary antibody. The intensity of bands was quantitated using Quantity One 4.5.0 image acquisition and analysis software (Biorad) and plotted. The values given are the average of five experiments ±SEM. ^∗^ statistically significant when compared to the control (*p* < 0.05).

The effect of 15(S)-HPETE on the production of CD31, biochemical marker of angiogenesis, by adipose endothelial cells was analysed. A significant decrease in cell associated CD31 was observed when cells were treated with 15(S)-HPETE when compared to control (Fig. 3A).

### Effect of 15(S)-HPETE on the production of VEGF

In order to study the molecular mechanism involved in the angiostatic effect of 15(S)-HPETE in adipose tissue, the influence of 15(S)-HPETE on the production of VEGF by endothelial cells derived from adipose tissue was studied. Analysis by ELISA showed about 55% decrease in the level of VEGF secreted into the medium on treatment with 15(S)-HPETE when compared to the control (Fig. 3B). Further analysis of VEGF by Western blotting confirmed decrease in the production of VEGF in cells upon treatment with 15(S)-HPETE when compared with the control (Fig. 3C).

### Effect of 15(S)-HPETE on Akt

**Figure 4:**
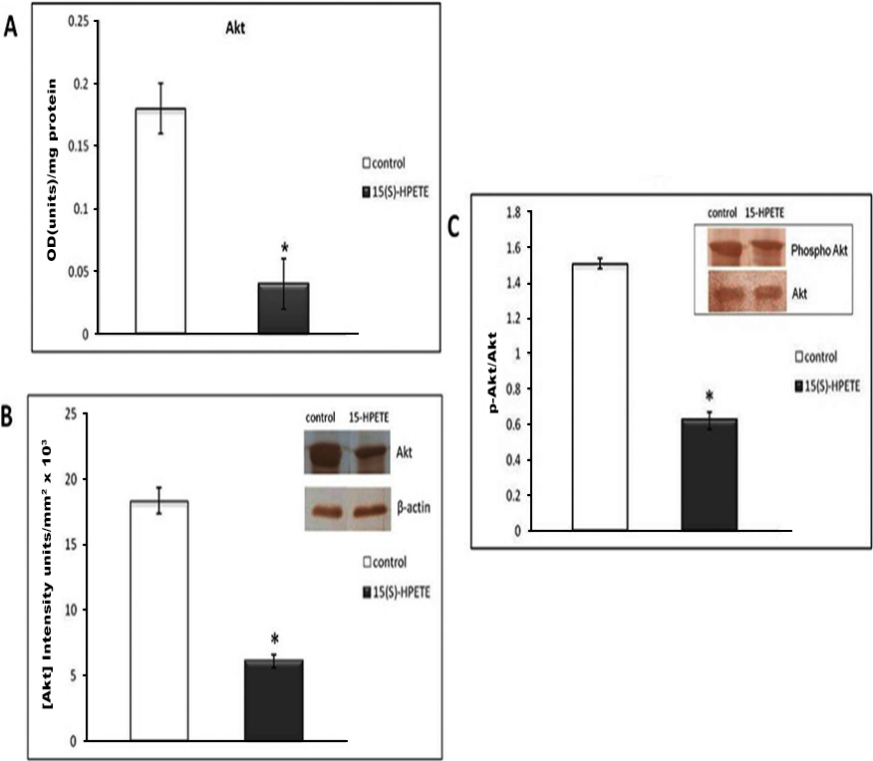
Effect of 15(S)-HPETE on Akt in adipose endothelial cells. Endothelial cells derived from adipose tissue were treated with MCDB 131 medium containing 0.1 µM 15(S)-HPETE in culture for 48 h. Cells treated with MCDB 131 medium containing 0.1% alcohol served as control. Cell layer was harvested and cell lysate containing equivalent amount of protein was subjected to ELISA (A) and Western blot analysis (B) using anti-Akt as primary antibody and anti-mouse IgG-HRP as secondary antibody. The intensity of bands was quantitated using Quantity One 4.5.0 image acquisition and analysis software (Biorad) and plotted. (C) Stromovascular tissues derived from adipose tissue were maintained in culture in MCDB 131 medium supplemented with 15(S)-HPETE (0.1 µM) for 48 h. The activation of Akt was examined by immunoblot analysis. Equivalent amount of Akt as determined by ELISA was immunoprecipitated from the tissue lysate and subjected to Western blot and probed with anti-phospho serine. The intensity of bands was quantitated and plotted as ratio of p-Akt:Akt. The values given are average of five experiments ±SEM. ^∗^ statistically significant when compared to control (*p* < 0.05).

Akt is reported to be a central signaling molecule in regulating angiogenesis. We examined the effect of 15(S)-HPETE on Akt in endothelial cells. The level of Akt was significantly decreased in cells treated with 15(S)-HPETE compared with the control. There was about 75% decrease in the level of Akt in cells treated with 15(S)-HPETE when compared to control (Fig. 4A). Further analysis by Western blotting confirmed the decreased production of Akt by adipose endothelial cells upon treatment with 15(S)-HPETE (Fig. 4B). The effect of 15(S)-HPETE on the activation of Akt was studied by analysing phosphorylated Akt by Western blot using stromovascular tissue derived from adipose tissue. In stromovascular tissue treated with 15(S)-HPETE, there was a significant decrease in the level of phosphorylated Akt as compared to control (Fig. 4C) suggesting that 15(S)-HPETE inhibited the activation of Akt in stromovascular fraction of adipose tissue.

### Effect of 15(S)-HPETE on apoptosis of endothelial cells

**Figure 5:**
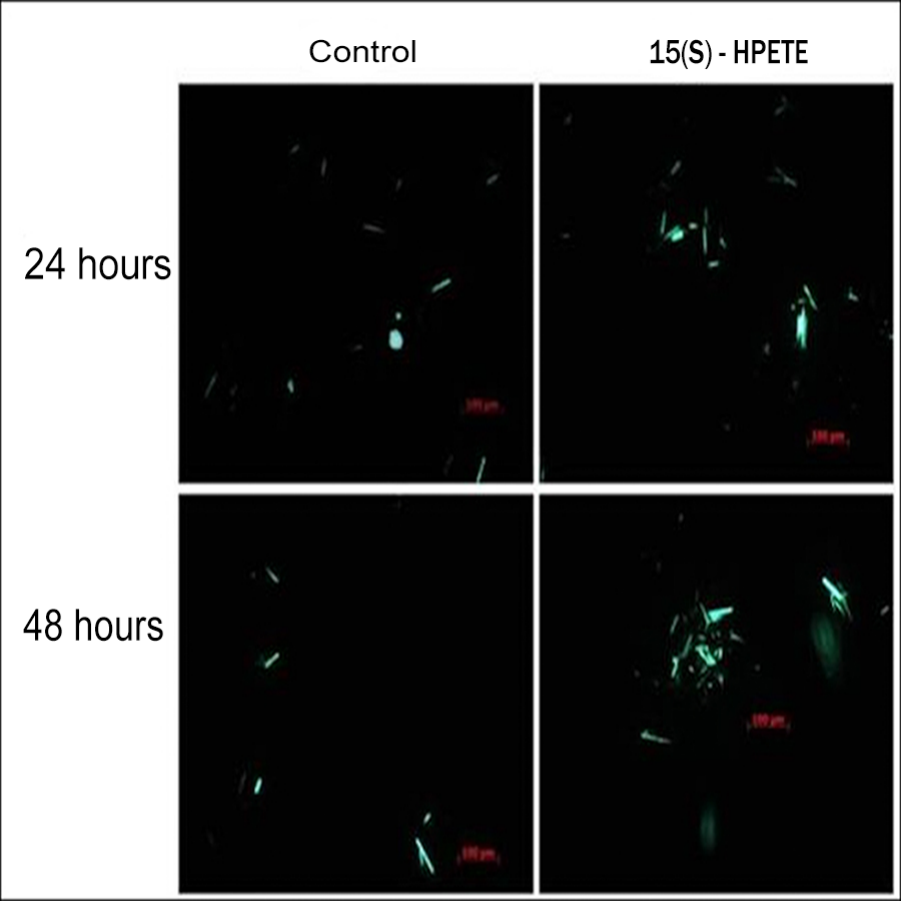
DAPI staining of adipose endothelial cells. Endothelial cells were isolated from adipose tissue and maintained in culture in MCDB 131 medium supplemented with 0.1 µM 15(S)-HPETE. Untreated cells served as control. Changes in the nucleus associated with apoptosis such as nuclear fragmentation (indicated by arrows) were seen in cells treated with 15(S)-HPETE.

To study whether the inhibition of angiogenesis by 15(S)-HPETE was due to apoptosis of endothelial cells, DAPI staining was performed. Significant nuclear staining of apoptotic cells was found in cells treated with 15(S)-HPETE when compared with the control indicating that 15(S)-HPETE induced apoptosis in adipose endothelial cells (Fig. 5).

### Effect of 15(S)-HPETE on Caspase-3

**Figure 6:**
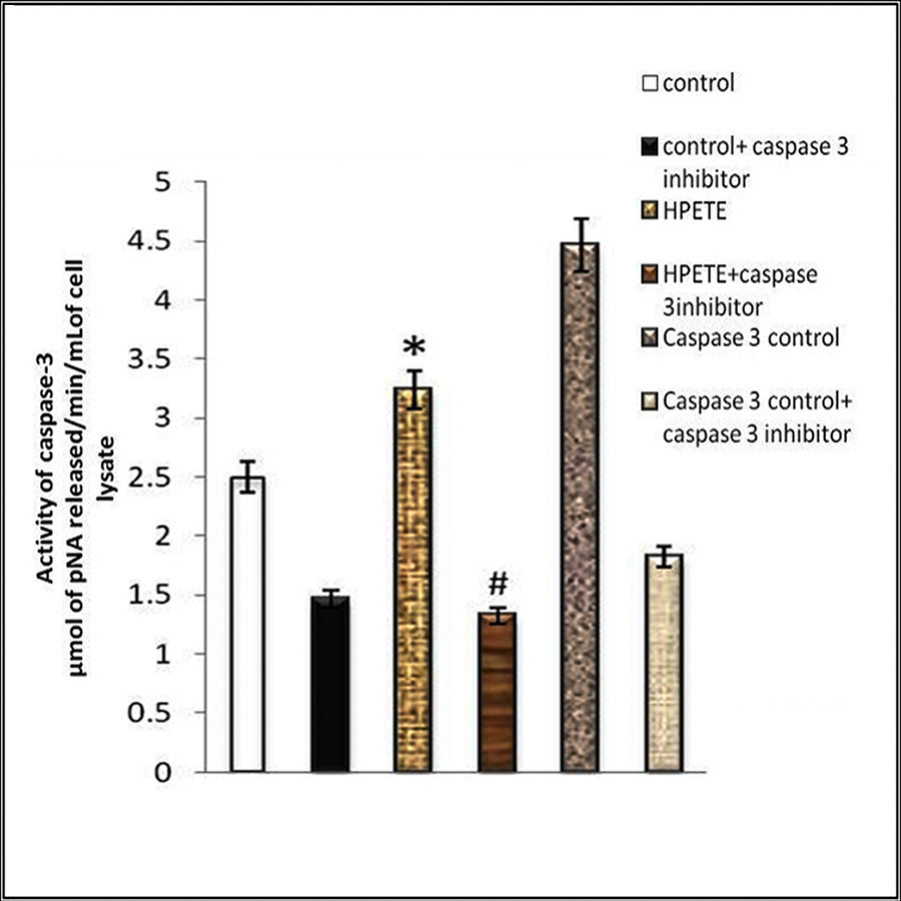
Effect of 15(S)-HPETE on Caspase-3 activity. Endothelial cells derived from adipose tissue were maintained in culture in MCDB 131 medium treated with 15(S)-HPETE (0.1 µM) for 24 h. After treatment, the cell layer was harvested and the activity of caspase-3 in the cell lysate was assayed by measuring the intensity of colored product *p*-nitroaniline formed by the action of caspase on peptide conjugate–substrate DEVD-*p*-nitroanilide. The intensity was measured by using a microplate reader at 405 nm. The values given are average of five experiments ±SEM. ^∗^ statistically significant compared to control. # statistically significant compared to 15(S)-HPETE treatment alone (*p* < 0.05).

As caspases play an important role in programmed cell death and is a key executioner of apoptosis, the activity of caspase-3 in endothelial cells treated with 15(S)-HPETE was determined. Results showed that the activity of caspase-3 was significantly higher in cells treated with 15(S)-HPETE than in the control cells suggesting that 15(S)-HPETE induced activation of caspase-3 in endothelial cells derived from adipose tissue (Fig. 6).

### Production of Bcl-2 and Bax in endothelial cells following 15(S)-HPETE stimulation

**Figure 7:**
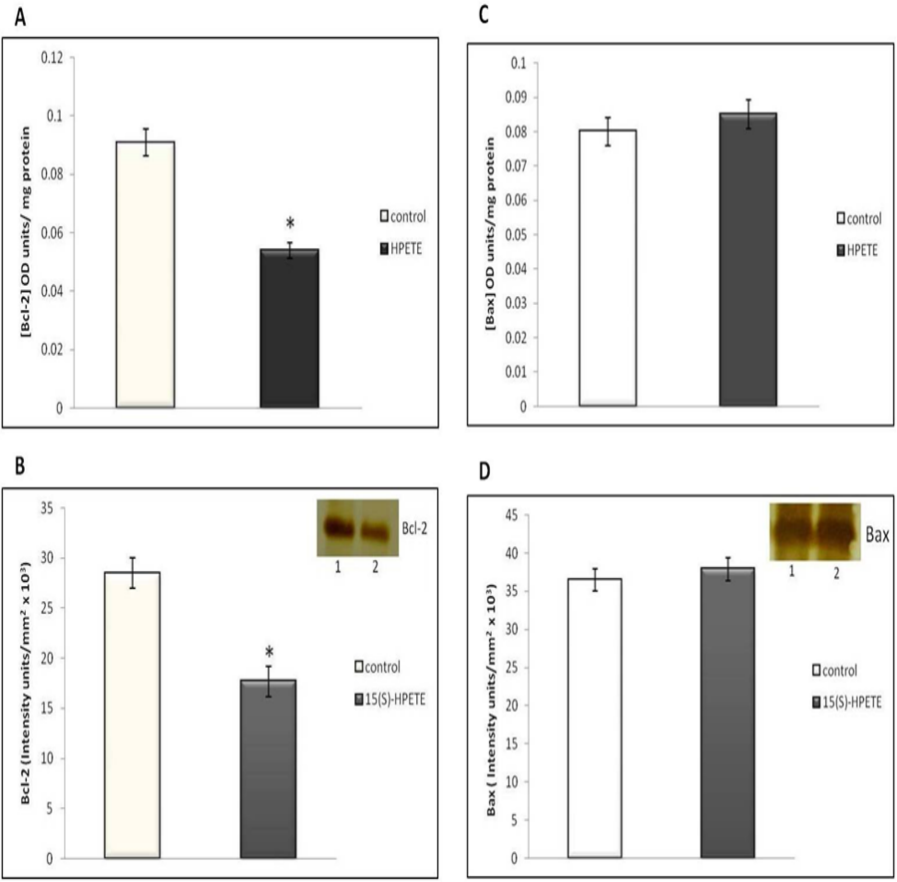
Effect of 15(S)-HPETE on Bcl-2 and Bax in adipose endothelial cells. Endothelial cells derived from adipose tissue were maintained in culture in MCDB 131 medium supplemented with 15(S)-HPETE (0.1 µM) for 48 h. Untreated cells served as control. (A) After treatment, the cell layer was harvested and the cell lysate was subjected to ELISA to estimate Bcl-2. (B) The levels of Bcl-2 was also determined by Western blot analysis using anti-Bcl-2. The intensity of bands was quantitated using Quantity One 4.5.0 image acquisition and analysis software (Biorad) and plotted. The values given are average of five experiments ±SEM. ^∗^ statistically significant compared to control (*p* < 0.05). (C) Cell lysate was subjected to ELISA to estimate Bax. The values given are average of five experiments ±SEM. There was no statistically significant difference between control and 15(S)-HPETE treated group (*p* > 0.05). (D) The level of Bax was also analysed by Western blot analysis using anti-Bax. The intensity of bands was quantitated. There was no significant difference between control and 15(S)-HPETE treated group (*p* > 0.05).

Bcl-2 is an anti-apoptotic molecule and Bax is pro-apoptotic. Bax/Bcl-2 levels determine the activation of caspase-3. Therefore, we studied whether 15(S)-HPETE has any effect on the production of Bcl-2 and Bax in endothelial cells. Endothelial cells in culture were treated with 15(S)-HPETE and the levels of Bcl-2 and Bax in the cell layer was analysed. 15(S)-HPETE significantly decreased the production of Bcl-2 in adipose endothelial cells when compared to control (Figs. 7A and 7B). But there was no significant difference in the level of Bax in cells treated with 15(S)-HPETE compared to control suggesting that 15(S)-HPETE-induced apoptosis in endothelial cells does not involve the activation of Bax (Figs. 7C and 7D).

### Discussion

15-Lipoxygenase enzymes play a role in regulating inflammation associated with obesity. 15-LOX metabolites can have both pro- and anti-inflammatory effects and thus it appears to be important in modulating adipocyte function in obese conditions (Iyer et al., 2010). Here we focused on the codependence of inflammation and angiogenesis in adipose tissue by studying the role of 15-LOX metabolites on adipose tissue-angiogenesis. The pro-angiogenic effect of 15(S)-HETE in adipose tissue was already reported (Soumya et al., 2013). The present study was designed to elucidate the effect of 15(S)-HPETE, precursor of 15(S)-HETE, on angiogenesis in adipose tissue and we found that 15(S)-HPETE inhibits angiogenesis in adipose tissue.

The anti-angiogenic effect of 15(S)-HPETE was evidenced by decreased endothelial sprouting from adipose tissue explants, inhibition of formation of tube-like structures in cultured endothelial cells derived from adipose tissue and decreased production of CD31 and VEGF by endothelial cells on treatment with 15(S)-HPETE. Endothelial cell–cell adhesion is an important process contributing to the formation of new vessels (De Lisser et al., 1997). Decrease in the levels of endothelial cell adhesion molecule CD31 on treatment with 15(S)-HPETE may result in decreased formation of cell–cell contact and endothelial tube-like structures. VEGF, a key mediator of angiogenesis in adipose tissue, activates endothelial cells and stimulates neovascularisation. 15(S)-HPETE mediates its anti-angiogenic effect by downregulating the production of VEGF. Similar results showing decrease in VEGF-production by 15(S)-HPETE in human umbilical vein endothelial cells was reported (Soumya et al., 2012). In rabbit skeletal muscles, 15-LOX 1 is reported to inhibit VEGF- induced angiogenic effects (Viita et al., 2008).

The mechanism underlying the angiostatic effect of 15(S)-HPETE was further studied. Inhibition of angiogenesis may be due to the inhibition of endothelial cell-proliferation or by the induction of apoptosis in endothelial cells. Several lines of evidence suggest the anti-proliferative nature of 15(S)-HPETE. 15-LOX metabolites show differential effects on cell proliferation; 15(S)-HPETE decreases and 15(S)-HETE increases the cell number (Kiran Kumar et al., 1993). 15(S)-HPETE is reported to induce injury in endothelial cells (Ochi, Morita & Murota, 1992). Enhanced 15(S)-HPETE production during oxidant stress, induces apoptosis of bovine aortic endothelial cells (Sordillo et al., 2005). Here we examined whether the anti-angiogenic effect of 15(S)-HPETE was due to the induction of apoptosis in endothelial cells. The apoptotic effect of 15(S)-HPETE in endothelial cells was evidenced by DAPI positivity in cells treated with 15(S)-HPETE.

Withdrawal of cell survival factors induces apoptosis of cell. Akt is a cell survival signaling molecule which inhibits apoptosis by inactivating apoptotic proteins such as Bad, Bax and Procaspase-9 (Brumatti, Salmanidis & Ekert, 2010; Franke et al., 2003). The levels of Akt and its activation to phospho-Akt were decreased in cells treated with 15(S)-HPETE suggesting that one of the mechanisms contributing to the apoptotic effect of 15(S)-HPETE might be the decrease of cell survival signaling molecule, Akt.

The molecular mechanism of apoptotic effect of 15(S)-HPETE was further studied by analyzing the expression of apoptotic factors. It was reported that 15(S)-HPETE induced apoptosis in cancer cells by modulating the expression of Bcl-2 family proteins involving pro-apoptotic Bax and Bad or anti-apoptotic Bcl-2 and Bcl-x (Mahipal et al., 2007). Our study showed that the levels of Bcl-2 in adipose endothelial cells was decreased, while no significant change in the levels of Bax was observed in endothelial cells on treatment with 15(S)-HPETE. Bax/Bcl-2 ratio was found to be high in cells treated with 15(S)-HPETE. Increase in Bax/Bcl-2 ratio causes increase in the activity of caspase-3, major executioner of apoptosis (Salakou et al., 2007). Our results showed that 15(S)-HPETE induced activation of caspase-3 in adipose endothelial cells. 15(S)-HPETE has been reported to inhibit growth of leukemia cells by caspase -dependent apoptosis (Mahipal et al., 2007). Though we have not analysed these intermediate steps, it appears that higher relative ratio of Bax/Bcl-2 on treatment with 15(S)-HPETE associated with increase in activity of caspase-3 can cause apoptosis of cells. Regarding the molecular target of 15(S)-HPETE, cleavage of cellular proteins such as DNA repairing protein, Poly (ADP-ribose) polymerase [PARP] and cytoskeletal proteins by caspase-3 may lead to nuclear fragmentation and apoptotic events (Elmore, 2007). Though from the present investigations, the cellular target of 15(S)-HPETE is not clear, the earlier report that 15(S)-HPETE, incorporated into the phospholipids of endothelial cell membrane, induced cell injury by producing lipid peroxidation is relevant in this context (Ochi, Morita & Murota, 1992).

## Conclusion

The angiostatic effect of 15(S)-HPETE in adipose tissue is due to the induction of apoptosis of endothelial cells. 15(S)-HETE stimulates angiogenesis in adipose tissue whereas 15(S)-HPETE induces apoptosis of endothelial cells and inhibits angiogenesis. The relative levels of 15(S)-HETE and 15(S)-HPETE may contribute to the modulation of angiogenesis and plasticity in adipose tissue. Increased levels of 15(S)-HETE in adipose tissue may result in the expansion of adipose tissue by stimulating neovascularisation and the increased levels of 15(S)-HPETE may result in the regression of adipose tissue by inducing apoptosis.

## Supplemental Information

10.7717/peerj.635/supp-1 Supplemental Information 1 Effect of 15(S)-HPETE on CD31 HUVECs were maintained in culture in MCDB 131 medium supplemented with 0.1 µM 15(S)-HPETE for 48 h. Western blot analysis of CD31 was done.
