# Supplementary material for: 15-LOX metabolites and angiogenesis: angiostatic effect of 15(S)-HPETE involves induction of apoptosis in adipose endothelial cells
Source: PeerJ. 2014 Oct 21;2:e635. doi: 10.7717/peerj.635 (PMC4207198; doi:10.7717/peerj.635)
Supplement: Supplemental Information 1 — HUVECs were maintained in culture in MCDB 131 medium supplemented with 0.1 µM 15(S)-HPETE for 48 h. Western blot analysis of CD31 was done. [file peerj-02-635-s001.pdf]

Supplementary data

Effect of 15(S)-HPETE on CD31 in endothelial cells

To examine the effect of 15(S)-HPETE on the expression of CD31 in endothelial cells, Human Umbilical Vein endothelial cells (HUVECs) were maintained in culture supplemented with 15(S)-HPETE and the expression of CD31 was measured by Western blotting. The expression of CD31 was found to be decreased significantly in HUVECs treated with 15(S)-HPETE when compared to control.

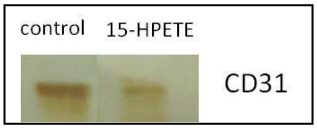

Effect of 15(S)-HPETE on the expression of CD31 HUVECs were maintained in culture in MCDB 131 medium supplemented with 0.1µM 15(S)-HPETE for 48 hours. Western blot analysis of CD31 was done.
